# 56326 Heart to Heart: An Interdisciplinary Community Collaboration to Address Health Disparities Through Cardiovascular Disease Risk Assessments in Underserved Urban Neighborhoods

**DOI:** 10.1017/cts.2021.745

**Published:** 2021-03-30

**Authors:** Michael E. Bales, Jifeng Zhu, Farid Aboharb, Neville Dusaj, Lior Shtayer, Venkatesh Balaji, Allegra Keeler, Christine A. Ganzer, Krista A. Ryon, the H2H Consortium, Brett J. Ehrmann, Julianne Imperato-McGinley

**Affiliations:** 1 Clinical and Translational Science Center, Weill Cornell Medicine; 2 Tri-Institutional MD-PhD Program, Weill Cornell Medicine, Rockefeller University, Memorial Sloan Kettering Cancer Center; 3 Joan & Sanford I. Weill Medical College, Weill Cornell Medicine; 4 Hunter-Bellevue School of Nursing, School of Health Professions, Hunter College, CUNY; 5 Department of Physiology and Biophysics, Weill Cornell Medicine; 6 Division of Primary Care of the Weill Cornell Physician Organization, Weill Cornell Medicine; 7 Department of Medicine & Clinical and Translational Science Center, Weill Cornell Medicine

## Abstract

ABSTRACT IMPACT: Leveraging partnerships with faith-based institutions and community centers in at-risk NYC neighborhoods, the H2H Program breaks down barriers to engaging with the medical establishment and addresses the increasing burden of diabetes and CVD risk factors in the most vulnerable individuals. OBJECTIVES/GOALS: Screening for modifiable risk factors is critical for cardiovascular disease (CVD) risk reduction. Low-income, urban communities often encounter barriers to care. Community-academic outreach partnerships are vital in addressing such disparities and promoting health equity and culturally targeted interventions among high-risk populations. METHODS/STUDY POPULATION: In 2010, the Weill Cornell Clinical and Translational Science Center along with Weill Cornell Medicine (WCM) and Hunter-Bellevue School of Nursing (HBSON) launched Heart to Heart (H2H), a community outreach program partnering with faith-based centers to offer free health screenings and education to some of New York City’s (NYC) most vulnerable communities. Participants work with undergraduate, nursing, medical and dietician students to complete a demographics and health questionnaire followed by vital signs and point-of-care blood testing. Participants then receive personalized health education, nutrition and lifestyle counseling by student volunteers, precepted by WCM Primary Care and HBSON faculty. Participants are provided information on local free or low-cost clinics as necessary for follow-up. RESULTS/ANTICIPATED RESULTS: To date H2H held 125 events and 5,952 screenings. Mean age of the participants was 54.3 (SD 39.6) and 3,682 (63.1%) were female. 74.2% identified as non-white. 42.1% were uninsured. 32.3% reported annual income of less than $20k. 18.3% of participants reported not having seen a doctor in the past year. 40.7% reported preexisting hypertension, of which 74.5% were on medication and 78% with sub-optimal control. 15.7% had been previously diagnosed with diabetes, of which 75.8% were on medication and 41.4% with sub-optimal control (HbA1c <7). 37.7% had been diagnosed with dyslipidemia previously, of which 47.4% were on medication and 62.1% with sub-optimal control. Screenings revealed, 56.9% had undiagnosed hypertensive blood pressures, 4.7% had an elevated HbA1c >6.5, and 49.2% had dyslipidemia. DISCUSSION/SIGNIFICANCE OF FINDINGS: H2H screening revealed significant cardiovascular health disparities, many of which were poorly controlled or newly discovered. Cross-institutional academic partnerships can empower communities with knowledge of their health status and help facilitate access to medical care to further address health risk factors.

